# Genome-wide identification and characterization of the CLASP_N gene family in upland cotton (*Gossypium hirsutum* L.)

**DOI:** 10.7717/peerj.12733

**Published:** 2022-01-03

**Authors:** Meijun Ji, Kangtai Sun, Hui Fang, Zhimin Zhuang, Haodong Chen, Qi Chen, Ziyi Cao, Yiting Wang, Allah Ditta, Muhammad Kashif Riaz Khan, Kai Wang, Baohua Wang

**Affiliations:** 1 School of Life Sciences, Nantong University, Nantong, Jiangsu, China; 2 Cotton Sciences Research Institute of Hunan/ National Hybrid Cotton Research Promotion Center, Changde, Hunan, China; 3 Plant Breeding and Genetics Division, Nuclear Institute for Agriculture and Biology, Faisalabad, Pakistan

**Keywords:** *Gossypium hirsutum*, CLASP_N, Gene family, Fiber development

## Abstract

**Background:**

Cytoplasmic linker–associated proteins (CLASPs) are tubule proteins that can bind to microtubules and participate in regulating the structure and function of microtubules, which significantly affects the development and growth of plants. These proteins have been identified in *Arabidopsis*; however, little research has been performed in upland cotton.

**Methods:**

In this study, the whole genome of the CLASP_N family was analyzed to provide theoretical support for the function of this gene family in the development of upland cotton fiber. Bioinformatics was used to analyze the family characteristics of CLASP_N in upland cotton, such as member identification, sequence characteristics, conserved domain structure and coevolutionary relationships. Real-time fluorescent quantitative PCR (qRT-PCR) was used to clarify the expression pattern of the upland cotton CLASP_N gene family in cotton fiber.

**Results:**

At the genome-wide level, we identified 16 upland cotton CLASP_N genes. A chromosomal localization analysis revealed that these 16 genes were located on 13 chromosomes. The motif results showed that all CLASP_N proteins have the CLASP_N domain. Gene structure analysis showed that the structure and length of exons and introns were consistent in the subgroups. In the evolutionary analysis with other species, the gene family clearly diverged from the other species in the evolutionary process. A promoter sequence analysis showed that this gene family contains a large number of cis-acting elements related to a variety of plant hormones. qRT-PCR was used to clarify the expression pattern of the upland cotton CLASP_N gene family in cotton fiber and leaves, and *Gh210800* was found to be highly expressed in the later stages of fiber development. The results of this study provide a foundation for further research on the molecular role of the CLASP_N genes in cotton fiber development.

## Introduction

Upland cotton is the most important natural fiber crop in the world (Chen et al., 2007). There are four cultivated species, including the two tetraploid species *Gossypium hirsutum* (upland cotton) and *G. barbadense* (sea island cotton) and the two diploid cotton species *G. herbaceum* and *G. arboreum* (Alkuddsi et al., 2013). Upland cotton accounts for more than 95% of the world’s cotton fiber production (Song et al., 2019). In contrast, sea island cotton (*G. barbadense*) produces higher-quality fiber but lower yields (Shi et al., 2015). Although fiber quality has been greatly improved in recent decades by conventional cultivar breeding programs, the demand for higher fiber quality in upland cotton has increased with the development of the modern textile industry and the need for diverse upland cotton products. The fiber length (FL), fiber strength (FS), fiber length uniformity (FU), fiber elongation (FE), and micronaire value (MIC) are important indicators for evaluating fiber quality (Wang et al., 2016a; Wang et al., 2016b), while maturity and fiber length are important factors that affect spinning (Kelly et al., 2019). As single-celled trichomes formed by ovule epidermal cells, cotton fibers are outstanding model systems to study plant cell elongation and cell wall and cellulose biosynthesis (Kim & Triplett, 2001). Approximately one-third of epidermal cells differentiate into spinnable fibers. The development process of cotton fiber includes the primary stage, elongation stage, secondary wall biosynthesis stage and mature stage. These four stages are distinct but overlapping (Kim & Triplett, 2001). In the elongation phase, fiber cells rapidly elongate and gradually reach their maximum length (Fang et al., 2014). In cultivated varieties of cotton, seed trichomes are composed of long lint fibers that are easily detached from the seed and fuzz fibers that are tightly attached to the seed (Applequist, Cronn & Wendel, 2001). In addition, fuzz fibers cannot be twisted many times during spinning. Therefore, long fibers should be used because they are easier to twist. Cotton fiber is a highly elongated and thickened single cell. The growth of cotton fiber cells undergoes stage differentiation, including the synthesis of primary wall in the elongation stage and the synthesis of cellulose in the thickening stage of the secondary wall. It is worth noting that the deposition of high ratio of cellulose in mature cotton fibers makes the fibers have good hardness and strength. This process is of great significance in nature and industry (Tang & Liu, 2017). Therefore, the elongation period and the thickening of the secondary cell wall are critical periods for the development of cotton fibers, which are worthy of further study.

In eukaryotes, microtubules and microtubule-associated proteins control cell growth and development by organizing microtubule arrays and spatial aspects (Hamada, 2014). Studies have shown that CLASPs mediate the function of *Arabidopsis* microtubules in response to mechanical stress on different spatial scales. This protein responds to different degrees of tissue-scale mechanical stress in the cotyledon epidermis by regulating the organization of microtubules (Eng et al., 2021). At present, many types of microtubule binding protein families have been discovered. The microtubule-binding proteins of these families affect microtubule organization through various functions, including regulating the microtubule dynamic properties, microtubule interactions, and microtubule cleavage (Kong et al., 2015). Cytoplasmic linker–associated proteins (CLASPs) are microtubule-binding proteins that can regulate the structure and function of microtubules (Pietra et al., 2013) and bind to microtubules and cytoplasmic connexins. Because it binds to the positive end of microtubules, CLASP is also called a positive tracer protein (Cleavage Akhmanova & Hoogenraad, 2005). This protein contains a typical conserved domain of the CLASP_N terminus, and some of the proteins also contain HEAT repeat domains (Sedbrook, 2004). The CLASP_N terminus usually contains two TOG or TOG-like (TOGL) domains and a basic serine-rich domain (Caroline et al., 2014). In eukaryotes, this protein regulates microtubule dynamic activity through these functional domains. There are few reports on the function of CLASP in plants, and most are based on the model plant *Arabidopsis thaliana*. There is only one CLASP gene in the *Arabidopsis* genome (Ambrose et al., 2007). Microtubules are the material transport tracks in cells, and damage to microtubules will hinder the material transport in cells (Gardiner, 2013). The arrangement of periplasmic microtubules is related to fiber cell elongation and secondary wall biosynthesis (Sumino et al., 2012). Studies have shown that the CLASP genes are related to the growth of root meristems, the elongation of hypocotyl cells, and leaf epidermal hair branching (Laryssa, Katherine & Geoffrey, 2020). Cotton fibers are single cells derived from elongated cells of the ovule epidermis. It is speculated that cotton fiber may have a similar regulatory mechanism to the development of the *Arabidopsis* epidermal hair with similar structural (Le & Ambrose, 2018). Since related genes have been identified in *Arabidopsis*, it is speculated that the CLASP_N gene family may play a role in the development of cotton fiber, *e.g.*, Zhu et al. (2018) found that *GhCLASP* is mainly expressed in stem fibers and developing fibers. Based on previous studies, we used the latest upland cotton reference genome to complete the genomic identification of the CLASP_N gene family. At the same time, we carried out chromosome mapping, multisequence alignment, and evolutionary tree construction for the genes of the family and motif prediction and gene structure identification of the CLASP_N gene family genes. This research will help reveal the molecular role of the *CLASP_N* genes in cotton fiber development.

## Materials & Methods

### Cotton material

The public upland cotton germplasm PD94042, which is characterized by improved fiber maturity and high yield potential (May, 1999), was used in this research. First, seeds with a full grain and similar sizes were selected. Seeds of PD94042 were planted in a field at Nantong University, and flowers were tagged before anthesis. The leaves of the one-leaf stage and the fibers at 17 and 21days post anthesis (DPA) were collected, and RNA was extracted for RT-qPCR validation.

### Identification and sequence analysis of CLASP_N family members in upland cotton

Whole genome data of upland cotton were downloaded from the JGI plant genome database (https://phytozome-next.jgi.doe.gov/info/Ghirsutum_v2_1) (Chen et al., 2020). The Hidden Markov Model (HMM) database (Protein families database of alignments) and Pfam database (http://pfam.xfam.org/) (Finn et al., 2016) were used to download the seed file PF12348 of the *CLASP_N* genes. After the screening, the protein sequences were uploaded to the online software Pfam, the Simple Modular Architecture Research Tool database (SMART, http://smart.embl-heidelberg.de) (Letunic, 2017) and the Conserved Domain database (CDD, https://www.ncbi.nlm.nih.gov/Structure/cdd/cdd.shtml), and sequence alignment and analysis were carried out to remove unannotated genes and redundant sequences (Marchler-Bauer et al., 2015). The gene IDs containing the CLASP_N special domain and its corresponding sequence were obtained. The functional annotation and GO annotation information of each gene was searched through the websites of https://jgi.doe.gov/ and http://geneontology.org/ respectively.

### Chromosome location and collinearity analysis

The gff3 annotation file of the genome of upland cotton was used to extract biological information, such as the position and structure of CLASP_N family members. Mapchart software (Voorrips, 2002) was used to analyze and demonstrate the chromosomal positions of the family genes. AI (Adobe Illustrator CS6) software was used to beautify the chromosome position map. To reveal the collinearity of the CLASP_N family among upland cotton species, all protein sequences in upland cotton were BLAST searched and compared using MCSCANX software (Wang et al., 2012), and collinear genes were found in the whole genome. The collinearity of genes in the gene family was disclosed, and the gene family circle was drawn using MCSCANX software with special annotation.

### Gene structure and motif analysis

The location information of all *CLASP_N* genes, including the exons, introns, and UTR location information on the chromosome, was available in the reference genome of the upland cotton database. A gene structure diagram (Hu et al., 2015) was drawn using the online website GSDS (http://gsds.cbi.pku.edu.cn/). MEME software (http://meme-suite.org/) (Bailey et al., 2006) was applied to carry out a motif analysis based on the protein sequences of *CLASP_N* genes. The exon-intron structure of the gene family was analyzed with the online software GSDS (http://gsds.cbi.pku.edu.cn/). TBTools was used to combine and visualize the analyzed sequence alignment results files, exon-intron structure files, and conservative domain files (Zhang, Raikhel & Hicks, 2013).

### Construction of phylogenetic tree of CLASP_N family members

For the identified *CLASP_N* gene members, the protein sequences of upland cotton, *Arabidopsis thaliana*, rice (*Oryza sativa*), tomato (*Solanum lycopersicum*), and corn (*Zea mays*) were extracted using HMMER 3.0 and a BLASTP search. The extracted protein sequences were then compared with MEGA 7.0 software (Sudhir, Glen & Koichiro, 2016). A phylogenetic tree was then built based on the comparison results. The evolutionary tree was constructed with neighbor-joining method (Li et al., 2019). The Bootstrap repeat test (Replications) was set to 1,000. The online software Evolview (http://www.omicsclass.com/article/671) was used to beautify the evolutionary tree.

### Cis-elements analysis of *CLASP_N* genes

To conduct the functional correlation analysis, 1.5 kb sequences located upstream of ATG of all *CLASP_N* genes were extracted to analyze the cis-elements of genes. The PlantCare (http://bioinformatics.psb.ugent.be/webtools/plantcare/html/) database (Lescot et al., 2002) was used to recognize and analyze the cis-acting elements of genes, and then they were visualized using TBTools.

### RNA extraction and quantitative real-time PCR (qRT-PCR) analysis

Total RNA was extracted from cotton fiber samples using a polysaccharide polyphenol complex plant RNA extraction kit (AIDLAB, Beijing, China) following the manufacturer’s procedure. A maximum of 1 µg total RNA was used to synthesize cDNA by HiScript QRT SuperMix (TAKARA, Beijing, China), and then qRT-PCR was performed with an Opticon thermocycler (CFXConnect Real-Time System; Bio-Rad, Hercules, CA) using SYBR Green PCR master mix (TAKARA, Beijing, China) according to the manufacturer’s instructions. Primer 5 was used to design the relevant primers needed for the PCR assay (Table S1). The PCR conditions were set as follows: 95 °C for 10 min, 40 cycles of 95 °C for 10 s and 60 °C for 30 s; and a 65–95 °C melt curve, which was analyzed to detect possible nonspecific amplification or primer dimers. The upland cotton histidine gene was used as a control for normalization between samples. The comparative threshold cycle method was used to calculate the relative transcript levels (Ma & Zhao, 2010).

## Results

### Genome-wide identification of CLASP_N family members

Using domain information (PF12348) obtained from domain prediction to identify members of the whole upland cotton genome, 35 genes were obtained. After comparing the results from the CDD, SMART, and other databases, a total of 16 genes were finally identified as belonging to the CLASP_N gene family. The gene information of upland cotton was searched by HMM and shown in Table S2. To maintain consistency, all upland cotton genes were temporarily named *Gh147000-Gh053074* according to the gene IDs. Functional annotation showed that most genes had similar functions, which were mainly related to spiral and folding. GO annotation indicated that the biological process involved in genes *Gh47000*, *Gh142700*, *Gh308900*, *Gh152100*, *Gh140100* and *Gh309600* are the regulation of lipid kinase activity, and the cellular components involved are spindle microtubules.

### Chromosomal localization and collinearity analysis in CLASP_N

**Figure 1:**
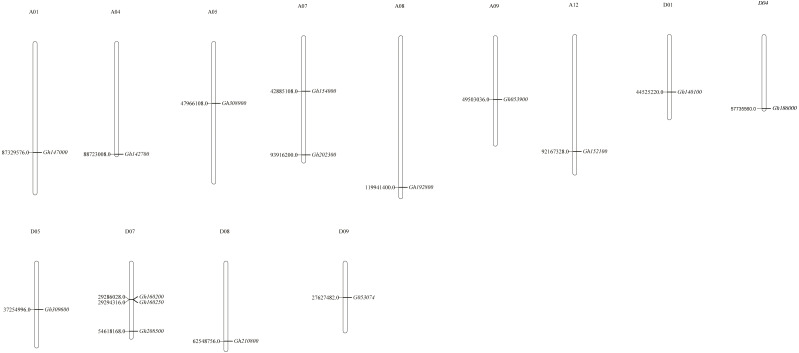
Chromosomal distribution of *CLASP_N* genes in upland cotton.

Chromosome positioning was carried out with the *CLASP_N* genes (Fig. 1). Our results indicated that the distribution of genes was uneven, with some chromosomes showing an overdistributed or tight arrangement in certain areas. Among them, 16 *CLASP_N* genes of upland cotton were located on 13 chromosomes and distributed on A01, A04, A05, A07, A08, A09, A12, D01, D04, D05, D07, D08 and D09. Most of them were located at the distal or proximal ends of the chromosomes.

**Figure 2:**
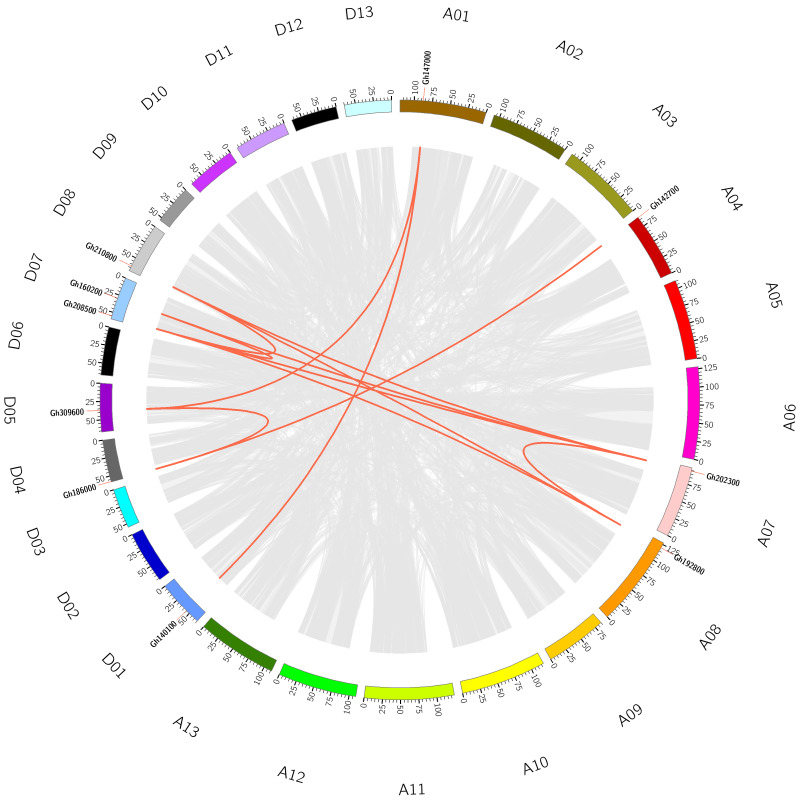
The collinearity of *CLASP_N* genes in the upland cotton. The gray lines represent collinear relationships within different genomes, and the red lines represent collinear gene pairs in the CLASP_N family.

To understand the evolutionary relationship of *CLASP_N* genes, collinearity analysis was conducted on *CLASP_N* genes of tetraploid upland cotton (Fig. 2), and there were 12 homologous gene pairs (Table S3). According to our study, there were many homologous gene pairs between D07 and D08, which indicated that different subgroups of upland cotton share a close evolutionary relationship. These results indicate that the gene family has undergone genomic rearrangement during the process of polyploidization.

### Multiple sequence alignment and gene structure analysis

The structures of 16 *CLASP_N* genes in upland cotton were detected by the GSDS website to further understand the evolution of the gene structure of the CLASP_N family in upland cotton. First, the exon-intron structure of the 16 *CLASP_N* genes in upland cotton was studied, and we found that the number of exons of *CLASP_N* genes in upland cotton was larger than that of introns. Among them, eight genes had 21 exons, seven genes had 22 exons, and only the *Gh160250* gene had nine exons. Most of the exons of *CLASP_N* genes were densely distributed.

**Figure 3:**
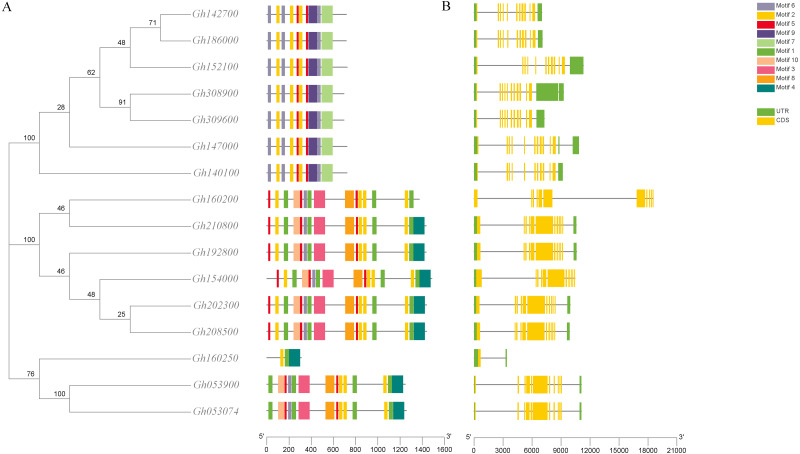
Phylogenetic tree, conserved motif and gene structure of CLASP_N protein in upland cotton. (A) Motif distribution of CLASP_N proteins. The conserved motifs in the CLASP_N proteins were identified with MEME software. Grey lines denote the non-conserved sequences, and each motif is indicated by a colored box numbered at the bottom. The length of motifs in each protein was presented proportionally. (B) The exon-intron structure of CLASP_N genes based on the evolutionary relationship. The yellow rectangle represents exons, the grey line represents introns, and the green rectangle represents UTR.

The analysis of the conserved motif of CLASP_N in upland cotton by MEME software (Fig. 3) showed that motif 2, motif 7, and motif 8 were all contained in the 6 subgroups of upland cotton, indicating that these 3 motifs were the most conserved motifs in the CLASP_N family. Motif 9 is unique to one group, while motif 4 is unique to two or three groups. However, the function of these conserved motifs remains to be studied. Generally, the gene structure analysis indicated that clusters in the same class presented relatively strong gene structure consistency. Moreover, the CLASPs of upland cotton in the same group usually contained similar motifs, indicating that they might play similar roles in the growth and development of upland cotton.

### Phylogenetic analysis of upland cotton CLASP_N

**Figure 4:**
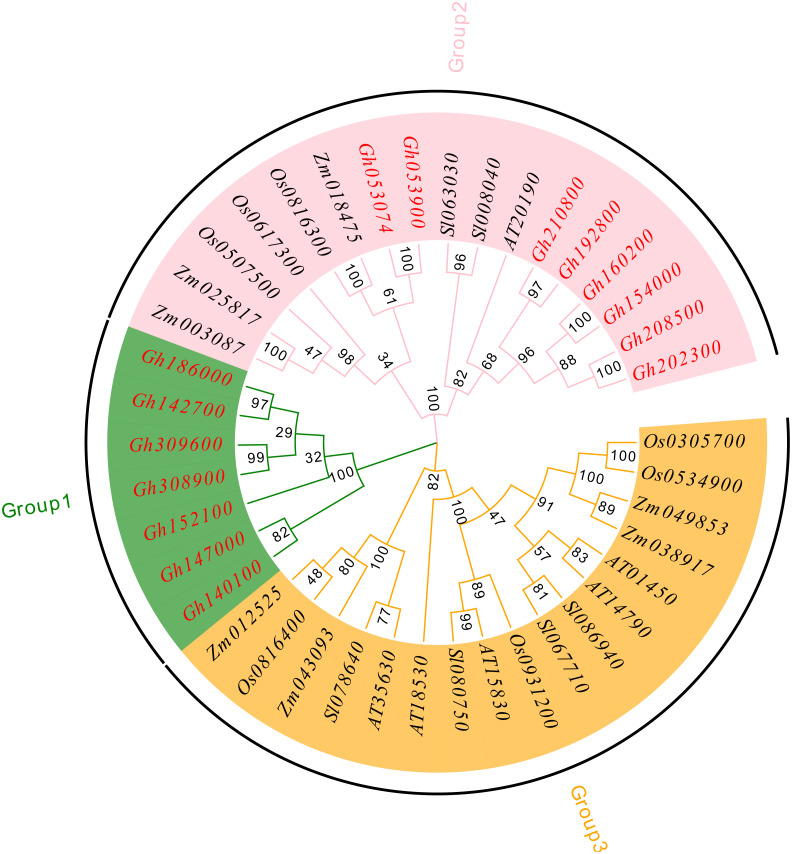
Phylogenetic tree of *CLASP_N* gene family members.

To understand the evolutionary relationship of the CLASP_N family, MEGA 7.0 software was used to compare and analyze the amino acid sequences of upland cotton (Chen et al., 2020), *Arabidopsis thaliana* (Cheng et al., 2017), tomato (*Solanum lycopersicum*) (Aflitos et al., 2015), rice (*Oryza sativa*) (Kawahara et al., 2013), and corn (*Zea mays*) (Wang et al., 2016a; Wang et al., 2016b) using the neighbor-joining method, and a phylogenetic tree was constructed (Fig. 4). According to our analysis and comparison results, the gene family is divided into three major groups, Group 1, Group 2 and Group 3, and each group is divided into several subfamilies. The results showed that the CLASP_N family members of upland cotton only existed in Group 1 and Group 2. Group 1 contained fewer family members and only contained family members of upland cotton. Genes from the same subgroup can be considered to have the same function. Group 2 and Group 3 contained almost the same number of family members. These results indicate genetic differentiation between *CLASP_N* genes in other species and upland cotton.

In addition, the upland cotton genes in Group 1 contained fewer motif types but were similar in type. The members of the upland cotton family in Group 2 contained a larger number of motifs, and the types of motifs contained in each member were generally similar. The results showed that all *CLASP_N* genes in the same subfamily had conserved motifs and similar gene structures, which could support the reliability of the phylogenetic classification.

### Cis-element analysis of promoter sequence

**Figure 5:**
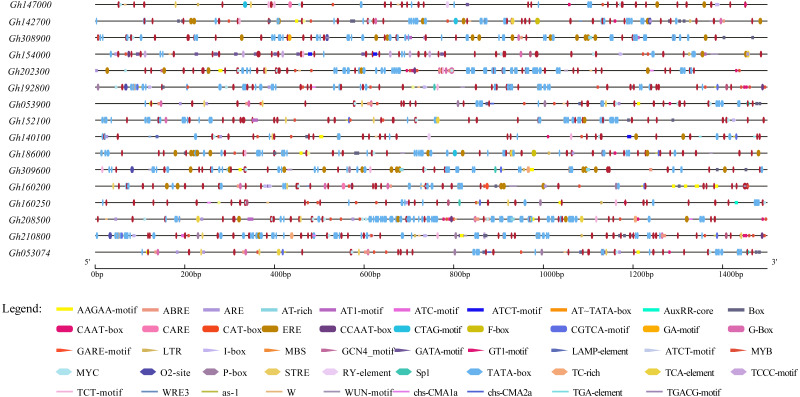
Homeopathic element analysis of upland cotton *CLASP_N* gene family.

To further study the regulatory mechanism of the upland cotton CLASP_N gene family in the abiotic stress response, 16 gene family sequences were extracted from the upland cotton genome information for a cis-acting element analysis (Table S4), and the results showed that 16 abiotic stress response elements were involved (Fig. 5). In addition to a large number of basic elements, CAAT boxes and TATA boxes, in the CLASP_N gene family of upland cotton, there are also cis-acting regulatory elements involved in the light response, such as G-box and light response (Zhang et al., 2020). The MYB binding site of the module part is a WRE3 element that participates in the photoreaction. MYB participates in the regulation of plant phenylpropane secondary metabolic pathways (Arce-Rodríguez, Martínez & Ochoa-Alejo, 2021). The WUN motif is a stress response element in plants (He et al., 2021), and ARE is a cis-acting regulatory element necessary for anaerobic induction (Mineo et al., 2021). The results of the study indicate that members of the CLASP_N gene family of upland cotton are involved in the process of responding to a variety of biotic and abiotic stresses.

### Expression pattern of *CLASP_N* genes at different times

**Figure 6:**
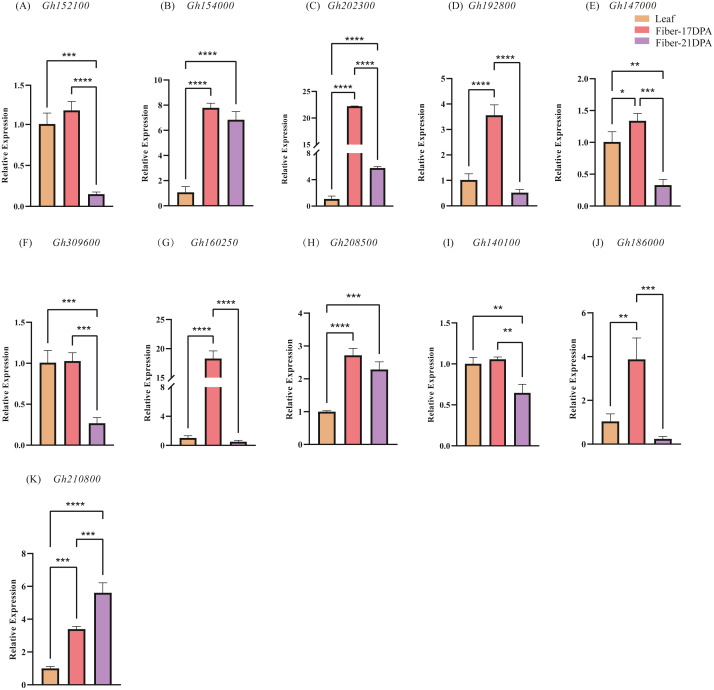
The relative expression levels of 11 candidate genes in upland cotton. Note: (A–E) belong to A subgenome, (F–K) belong to D subgenome. The asterisk at the top of the bar indicates the statistically significant difference between the various periods (* *P* < 0.05, ** *P* < 0.01, *** *P* < 0.001, **** *P* < 0.0001).

To further explore the expression changes in the *CLASP_N* genes in different tissures at different time, qRT-PCR was used to investigate the transcript levels of each *CLASP_N* gene at leaves, fibers of 17 DPA and 21 DPA, respectively, and we performed 3 biological repetitions and 2 technical repetitions (Table S5). Generally, most of the expression levels at fiber-21 DPA were lower than those at fiber-17 DPA (Fig. 6). The relative expression at fiber-21 DPA was significantly higher than at fiber-17 DPA only for *Gh210800*. The expression levels of fiber-21 DPA in *Gh152100*, *Gh192800*, *Gh147000*, *Gh309600*, *Gh160250*, *Gh140100*, and *Gh186000* were lower than that at one-leaf stage and fiber-17DPA. For *Gh154000*, *Gh202300*, *Gh208500*, and *Gh210800,* the expression level at both fiber stages was significantly higher than that at the one-leaf stage. The expression of *Gh210800* is significantly higher than that of the other genes, and its expression level increased with time past.

## Discussion

CLASPs are proteins that bind to the positive end of microtubules and have the ability to bind to both microtubule and cytoplasmic junction proteins. CLASPs generally contain two conserved domains: the HEAT repeat domain and the CLASP_N terminal domain. Some CLASPs contain only the CLASP_N structure. The N-terminus of most CLASPs contains two TOG or TOG-like (TOGL) domains, followed by a basic serine-rich domain. In vitro, this domain is sufficient for fission yeast Peg1/Cls1 to bind to the spatial regulation of microtubule (MT) lattice with high affinity (Al-Bassam et al., 2010), whereas Stu1, a CLASP essential for spindle integrity, additionally requires part of the TOGL2 domain (Yin et al., 2002). Stu1 in *Saccharomyces cerevisiae* is needed for the MTs in vivo. Depending on the stage of mitosis, Stu1 is also recruited to the spindle MTs through different mechanisms: in the middle stage, Stu1 directly binds to the MT lattice, while in the later stage, it is located indirectly in the middle of the spindle (Caroline et al., 2014). Similarly, in *S. pombe*, Cls1 localizes to the spindle midzone in anaphase (Caroline et al., 2014). In vivo and *in vitro* experiments have demonstrated that the HEAT domain can form helix structures, regulate the interactions between proteins and participate in the transport of intracellular substances (Groves et al., 1999). The CLASP_N terminal domain is a very conserved domain, which is the binding site of microtubules and has the stabilizing effect of regulating dynamic microtubules. In this study, a total of 16 members of the CLASP genes in upland cotton were identified. The proteins encoded by the genes varied in length, but all of them had the typical CLASP_N terminal domain. In vivo, CLASPs regulate only a subset of cellular MTs, which may be caused by specific CLASP localization (Al-Bassam et al., 2010). During interphase and mitosis of mammalian cells, CLASP1 localizes to the centrosome and to the plus ends of MTs (Maiato et al., 2003). Studies have shown that in *Arabidopsis thaliana*, the lack of function of *AtCLASP* inhibits the transport of growth hormone and other substances; therefore, *Arabidopsis* shows phenotypes similar to plants lacking growth hormone, such as short plant type, multiple root branching, slow plant development and short pod. Microtubules are channels for plant material transportation (Brandizzi & Wasteneys, 2013; Kakar et al., 2013; Zhang, Zhang & Zhao, 2013). How CLASPs in cotton regulate the involvement of microtubules in the transportation of intracellular substances remains to be further studied.

Due to differences in CLASP functional domains, their family members in upland cotton may have different functions. Recent cotton genomics and genetics development has enabled us to systematically study the *CLASP_N* genes and explore their potential functions in the biological clock (Wang et al., 2020). In the phylogenetic tree, the 16 upland cotton *CLASP_N* genes were divided into 3 subtypes (Fig. 3). Among them, *Gh160250*, *Gh053900*, and *Gh053074* belong to the third subgroup. The structure of the *Gh160250* gene is very different from that of the other genes. This difference in the evolutionary process may be related to subfunctionalization and natural selection of species. Due to the high collinearity of genes in subgroup A and subgroup D of the tetraploid upland cotton genome, 16 members of the *CLASP_N* genes in upland cotton had multiple pairs of collinearity genes (Chen et al., 2020). Homologous genes always have the same biological function in the evolution stage, and their exon-intron structure and motif distribution are the same (Altenhoff & Dessimoz, 2009).

An analysis of chromosome location and genome collinearity indicated that the hybridization of upland cotton A and D subgenomes involves gene amplification through tandem duplication and fragment duplication (Jackson & Chen, 2010). A high degree of collinearity occurs between the *CLASP_N* genes of At and the Dt subgenome of the tetraploid *G. hirsutum*. In this study, many of the 16 CLASP_N family members were homologous genes, indicating that upland cotton has undergone large-scale gene rearrangements at the genome level during speciation (Wang et al., 2018). For the 16 genes identified, 11 genes were extracted for qRT-PCR analysis. Comparison of gene expression in different tissues showed that the gene expression level in leaves is generally lower than that in fiber-17DPA; meanwhile, the expression level in leaves is higher than that of fiber-21DPA for 4 genes, whereas lower for the rest 7 genes. A comparison of the gene expression at fiber-17 DPA and fiber-21 DPA showed that the trend of gene expression level in A subgenome (Figs. 6A–6E) was basically similar to that of D subgenome (Figs. 6F–6J) with a decreasing trend in fiber-21 DPA comparing to fiber-17 DPA except for *Gh210800* (Fig. 6K), indicating that most of these genes played important roles in the early phase of fiber growth.

An analysis of the conserved domains showed that the *Gh053074* gene contained only motif 1, motif 2 and motif 4 while the rest of the genes contained more motifs. Among them, the structures of the two subgroups containing *Gh160200* and *Gh053900* were similar, indicating that they may have similar functions (Fig. 3). Systematic analysis and research can be carried out according to the subgroup classification on the gene expression pattern map. The analysis of the cis-acting elements of the promoters revealed that almost every gene has phytohormone response elements. Phytohormones play an important role in regulating plant growth and development. For example, the ABRE is an abscisic acid response element in plants that binds to transcription factors to promote or inhibit the expression of abscisic acid-induced genes. It has been confirmed in *Arabidopsis* that this element is related to the stress resistance of plants (Pan et al., 2020). Other elements related to adversity are also included in this research, *e.g.*, the cis-acting element involved in the MeJA response is CGTCA-Motif, and the cis-acting element involved in the low-temperature response is LTR. TCA-element is a cis-acting element involved in the salicylic acid response, TC-rich repeat is a cis-acting element involved in defense and stress responses, P-box is a gibberellin response element, and TGA-element is an auxin-responsive element. The homeopathic element involved in the MYB transcription factor-binding site under drought stress is MBS. The CGTCA motif is a cis-acting regulatory element involved in MeJA responsiveness. The regulation of MeJA during flower development involves various physiological processes, such as filament elongation, pistil development, anther development and anther dehiscence. Among a variety of plants, such as wheat, rice, and rape, MeJA plays an important role in regulating anther dehiscence. Therefore, plant hormone response elements in the promoter sequence of the *CLASP_N* genes may regulate the development of anthers in cotton. Elements involved in hormone regulation have been found in many promoter regions, including ABREs, CGTCA motifs, P-boxes, TCA elements, and TGA elements (Chen & Liu, 2019). These homeopathic elements likely play a very important role in the development of cotton. In summary, we can infer that *CLASP_N* genes in upland cotton may have a certain regulatory role under hormonal and adversity stress. Upland cotton fiber development can be divided into four stages, and many genes are involved in the development and regulation of fibers at each stage of fiber development. During the secondary thickening of the cotton fiber cell wall, the number of periplasmic microtubules increased significantly, and their arrangement direction was consistent with the direction of the newly formed fiber layer, whereas the synthesis of the cotton fiber cell secondary wall was related to the arrangement pattern of the periplasmic microtubules (Dixit & Cyr, 2004). Therefore, these genes may affect the development of cotton fiber by regulating the dynamic reorganization of microtubules and the change in the arrangement direction. According to previous studies, the *CLASP_N* genes can act on microtubules and cell membranes at the same time, which may be related to the transportation of intracellular substances (Yaffe, Stuurman & Vale, 2003). Previous work showed that CLASP_N family genes were located on the mitochondrial membrane. CLASPs may regulate microtubules, affect the structure, distribution or function of mitochondria in fiber cells, and participate in the regulation of fiber development. By regulating the orientation and dynamic distribution of microtubules, CLASPs participate in the regulation of fiber development, affects fiber elongation and secondary growth, and finally affects fiber quality.

## CONCLUSIONS

Based on genomic data of upland cotton, a genome-wide identification analysis of upland cotton CLASP_N family was performed, and 16 family members were identified. A comprehensive analysis of their phylogenetic relationships, gene structures, evolution processes, promoter sequences, and expression patterns revealed their contribution to cotton fiber quality. These results will facilitate further investigations of the molecular role of *CLASP_N* genes in cotton fiber development.

## Supplemental Information

10.7717/peerj.12733/supp-1 Supplemental Information 1 Primers of qRT-PCR for candidate genes

10.7717/peerj.12733/supp-2 Supplemental Information 2 Physicochemical information of the 16 *CLASP_N* genes in upland cotton

10.7717/peerj.12733/supp-3 Supplemental Information 3 Tandem repeat genes of CLASP_N family in upland cotton

10.7717/peerj.12733/supp-4 Supplemental Information 4 Analysis of cis-acting elements of 16 *CLASP_N* genes in upland cotton

10.7717/peerj.12733/supp-5 Supplemental Information 5 The Ct value of qPCR raw data

## Additional Information and Declarations

### Data Availability

The following information was supplied regarding data availability: The raw data is available in the Supplemental Files.
